# Parents’ and Students’ Perceptions of Telepractice Services for Speech-Language Therapy During the COVID-19 Pandemic: Survey Study

**DOI:** 10.2196/25675

**Published:** 2021-01-28

**Authors:** Joseph Hin Yan Lam, Stephen Man Kit Lee, Xiuli Tong

**Affiliations:** 1 Human Communication, Development, and Information Sciences Faculty of Education The University of Hong Kong Hong Kong

**Keywords:** eHealth, telepractice, speech and language pathology, user satisfaction, COVID-19, school-based service

## Abstract

**Background:**

The ongoing COVID-19 pandemic has resulted in the suspension of face-to-face classes and a considerable increase in the use of telepractice services in speech-language pathology. However, little is known about parents’ and students’ satisfaction with telepractice services and their preferences for different service delivery modes. These factors may affect therapy effectiveness and the future adoption of telepractice.

**Objective:**

We evaluated students’ and parents’ perceptions of telepractice efficacy and their preferences for different service delivery modes (ie, on-site practice vs telepractice). We also identified factors that affect parents’ and students’ preferences for different service delivery modes during the COVID-19 pandemic.

**Methods:**

A 19-question survey on telepractice satisfaction and preferences was administered to 41 Hong Kong Chinese students and 85 parents who received telepractice services from school-based speech-language pathologists during the COVID-19 class suspension period. In addition to providing demographic information and data on the implementation of telepractice services, all participants were asked to rate their perceptions of the efficacy of telepractice services and compare on-site practices to telepractice on a 5-point Likert scale (ie, 1=strongly disagree/prefer the use of on-site speech-language therapy services and 5=strongly agree/prefer the use of telepractice services).

**Results:**

Despite the fact that telepractice efficacy was highly rated by parents (95% CI 3.30-3.66) and students (95% CI 3.21-3.76), both groups believed that telepractice was less effective than on-site practices (parents: 95% CI 2.14-2.52; students: 95% CI 2.08-2.65). Moreover, parents preferred on-site practices over telepractice (95% CI 2.04-2.43), whereas students did not prefer one mode of practice over the other (95% CI 2.74-3.41). A significant association between telepractice efficacy and a preference for telepractice services was found only among the students (τ=.43, *P*<.001), not the parents (τ=.07; *P=*.44).

**Conclusions:**

Although telepractice is an acceptable alternative service delivery option for providing speech and language therapy services to school-aged individuals, speech-language therapists and parents must play a more proactive role in telepractice services to facilitate effective communication between clinicians and parents.

## Introduction

As of January 2021, over 90 million people have been infected with the SARS-CoV-2 virus. This has necessitated social distancing and school closures worldwide. As a result, telehealth (ie, the use of audio or videoconferencing technology to provide health care services) has received increasing attention. Telehealth care has been regarded as an alternative to face-to-face care in many countries [1,2]. Furthermore, speech-language pathologists have engaged in telepractice over the past 2 decades in various countries [3-6]. The efficacy of telepractice has been supported by scientific research on speech, language, voice, and fluency disorders across different age groups [7-9]. Additionally, telepractice has been deemed valid and effective by different professional organizations [10,11]. With the COVID-19 pandemic seriously disrupting the provision of speech and language therapy services, telepractice services have been increasingly adopted and regarded as the best option for delivering speech and language therapy during the pandemic [12,13].

Despite the increasing adoption of telepractice in schools, various stakeholders have held different beliefs about telepractice. Although several surveys have shown that school-based speech-language pathologists doubt the efficacy of telepractice, others have revealed a positive attitude after using telepractice services [12,14,15]. However, parents’ and children’s perceptions of telepractice are not well understood. A few studies have examined parents’ and students’ satisfaction with telepractice programs, but the findings have been mixed. In a pilot survey, 13 teachers and 8 parents from a remote school were highly satisfied with the progress brought about by telepractice [8]. Positive findings were also noted in parents’ and students’ responses to a survey on web-based speech and language interventions that were conducted by university clinics [8,16]. In contrast, an interview study of 5 parents raised concerns about poor telepractice engagement by students and ineffective communication between parents and clinicians in telepractice services [17]. These factors may lower people’s acceptance of school-based telepractice services [17]. Given the high rate of telepractice adoption in school settings during the pandemic [12,13], a survey study on parents’ and students’ satisfaction with telepractice could reveal the perceived efficacy of these services.

Perceived efficacy is an important measure in speech and language therapy for both on-site practices and telepractice, because it reflects the effectiveness of the therapy and students’ and parents’ motivations for undergoing the therapy [18,19]. The Davis’ Technology Acceptance Model also argues that perceived efficacy, which is based on perceived usefulness and convenience, influences the future adoption of technology [20]. Perceived efficacy can be reflected by people’s engagement with therapy sessions, which correlates with children’s treatment outcomes [21]. Moreover, the amount of therapeutic skills that families practice during their daily routine and the collaboration between clinicians and parents affect the generalization of treatment [22]. Therefore, investigating parents’ and students’ perceptions of telepractice efficacy and their involvement with telepractice and daily therapeutic practices are critical for evaluating treatment fidelity.

Previous studies have largely focused on students’ and parents’ satisfaction with research-oriented telepractice, but none have investigated clients’ and parents’ preferences for different modes of practice. Since service delivery modes have expanded during the pandemic, students’ and parents’ preferences for different delivery models are critical for designing a future service delivery model for school-based speech and language therapy services. Thus, in this study, we examined how clients’ therapy characteristics, including age, comorbidity, and parent support, influence their preferences for different modes of service. This information may inform speech-language pathologists about selecting appropriate students for telepractice services [10].

In summary, the following 3 research questions were addressed in this satisfaction survey study: (1) what are parents’ and students’ perceptions of telepractice efficacy; (2) do parents and students prefer on-site practices or telepractice; and (3) what are the critical factors that affect parents’ and students’ preferences for different service delivery modes?

## Methods

### Survey Design and Development

#### Survey Summary

We developed a web-based survey for both parents and students to evaluate school-based speech and language therapy practices in Hong Kong (see Multimedia Appendix 1). To meet internal clarity, construct, and content validity criteria, all survey questions were independently reviewed by 3 school-based speech-language pathologists. This review ensured that the survey’s wording, content, and question order were clear and appropriate. The survey questions were revised and finalized in accordance with the speech-language pathologists’ suggestions. All respondents completed the survey in about 10 minutes. Ethics approval was granted by the Human Research Ethics Committee of University of Hong Kong, and participants signed consent forms before completing the survey.

The survey for parents and students consisted of 4 sections, including (1) the implementation of telepractice, which consisted of 2 items; (2) telepractice efficacy, which consisted of 7 items for parents and 4 items for students; (3) the comparison between telepractice and on-site practice, which consisted of 6 items for parents and 5 items for students; and (4) demographics, which consisted of 5 items. All responses for sections 2 and 3 were based on Likert-type scale scores, which ranged from 1 (ie, strongly disagree) to 5 (ie, strongly agree).

#### Section 1: Implementation of Telepractice

The 2 items in this section assessed the amount of therapy students received and how frequently students used telepractice services during the COVID-19 class suspension period.

#### Section 2: Telepractice Efficacy

The 7-item survey for parents included questions about whether telepractice was effective in enhancing their child’s language skills, meeting their child’s needs, engaging with their child, and providing satisfaction with the amount of therapy their child received (Cronbach α=.94). The 4-item survey for students included questions about whether telepractice services met their needs and whether they enjoyed telepractice services (Cronbach α=.84).

#### Section 3: Comparison Between Telepractice and On-site Practice

The 6-item parent survey included questions about whether telepractice services for speech therapy provided better communication than on-site speech and language therapy. There were also questions regarding the implementation of home therapy practices (Cronbach α=.89). The 5-item student survey included questions about whether students learned better language skills and exhibited better engagement with on-site practices than with telepractice (Cronbach α=.88).

#### Section 4: Demographics

The 4 items in this section were used to collect information on each student’s grade, gender, special education needs status, and family income.

### Participants

From July to August 2020, 85 parents (ie, 75 mothers and 10 fathers) and 41 students (ie, 7 girls and 34 boys) participated in our web-based survey. Based on the last 4 digits of participants’ telephone numbers, 27 families participated in both the parent and student surveys. These 27 families accounted for the 31% (27/85) of parents and 65% (27/41) of students who participated. The families who responded to both the parent and student questionnaires represented students from Grades 1-7 (parents’ questionnaire: median=Grade 3; students’ questionnaire: median=Grade 4). In terms of students’ comorbidities in the parent survey, the most prevalent special educational needs subtype was autism spectrum disorder (53/85, 62%), followed by attention deficit/hyperactivity disorder (33/85, 38%), specific learning difficulties (20/85, 23%), intellectual disabilities (3/85, 3%), hearing impairment (2/85, 2%), visual impairment (1/85, 1%), and physical disabilities (1/85, 1%). Additionally, 12% (11/85) of students had no comorbidities except for speech and language disorders. In terms of students’ comorbidities in the student survey, the most prevalent special educational needs subtype was autism spectrum disorder (24/85, 58%), followed by attention deficit/hyperactivity disorder (15/41, 36%), specific learning disorders (6/41, 14%), intellectual disabilities (1/41, 2%), and visual impairment (1/41, 2%). Additionally, 21% (9/41) of students had no comorbidities except for speech and language disorders. Around half of the participants (parents’ survey: 42/85, 49%; students’ survey: 22/41, 53%) had an average monthly family income that fell below the median for average household income (ie, around US $3290).

To achieve a Cronbach α value of .05 and a moderate effect size (ie, Cohen *d*=0.5), a statistical power of .99 and .86 was needed for 85 parents and 41 students, respectively. This was determined by using G*Power 3 software (G*Power Team) [23]. In addition, good quality results can be obtained by performing a factor analysis on samples with at least 50 people or samples with a factor loading value of >.60 [24].

## Results

### Implementation of Telepractice

Most students reported that they had fewer than 5 telepractice sessions during the pandemic (parents’ survey: 73/85, 85%; students’ survey: 31/41, 75%). In terms of session frequency, the most common amount of therapy was 1 session per month (parents’ survey: 35/85, 41%; 36%; students’ survey: 15/41, 36%), followed by 1 session per 2 weeks (parents’ survey: 25/85, 29%; students’ survey: 15/41, 37%), and 1 session per week (parents’ survey: 21/85, 24%; students’ survey: 12/41, 29%).

### Telepractice Efficacy

Parents and students had positive views of the efficacy of telepractice with respect to their understanding of the treatment goals (parents: mean 3.48, SD 0.84; 95% CI 3.30-3.66; students: mean 3.49, SD 0.87; 95% CI 3.21-3.76) and the ability of telepractice services to meet the needs of students (parents: mean 3.24, SD 1.03; 95% CI 3.01-3.46; students: mean 3.49, SD 0.84, 95% CI 3.22-3.75). Based on the parents’ responses, parents had positive views of students’ enjoyment of telepractice services (mean 3.29, SD 1.14; 95% CI 3.05-3.54) and the ability of telepractice services to enhance students’ language abilities (mean 3.33, SD 1.01; 95% CI 3.11-3.55). Based on the students’ responses, students had a neutral view of telepractice efficacy with regard to (1) enjoyment (mean 3.32, SD 1.08; 95% CI 2.98-3.66) and (2) language ability enhancement (mean 3.29, SD 0.96; 95% CI 2.99-3.59). Independent 2-tailed sample *t* tests revealed that there were no significant differences in the above views between parents and students (enjoyment: *P*=.92; understanding of treatment goals: *P*=.97; meeting students’ needs: *P*=.18; language ability enhancement: *P*=.85). In addition, parents held a positive view of the progress that students made during telepractice services (mean 3.35, SD 0.96; 95% CI 3.15-3.56) and a neutral view of the amount of therapy that students received (frequency: mean 2.99, SD 1.04; 95% CI 2.76-3.21; amount of therapy: mean 3.21, SD 1.03; 95% CI 2.99-3.43).

### Factors That Affected Telepractice Efficacy

Our Spearman rank-order correlation analysis showed that there were no significant correlations between student grade and perceived telepractice efficacy (parents: ρ=0.03; *P=*.76; students: ρ=0.07; *P=*.65). The Bayes factor (BF) was computed to evaluate whether the evidence supported the null hypothesis over the alternative hypothesis. BF_01_ values of >3 and >10 indicated moderate and strong support, respectively, for the null hypothesis [25]. Strong evidence that supported the null hypothesis (ie, no correlation between grade and telepractice efficacy) was found in the parent group (BF_01_=11.34), whereas moderate evidence that supported the null hypothesis was found in the student group (BF_01_=7.84).

### Comparison Between Telepractice and On-site Practice

Students’ enjoyment of telepractice services and on-site services was comparable, based on the students’ responses (mean 2.93, SD 1.06; 95% CI 2.59-3.26). However, students’ enjoyment of telepractice services was lower in the parents’ responses (mean 2.76, SD 1.02; 95% CI 2.54-2.98). Furthermore, telepractice was rated lower than on-site practice in terms of treatment effectiveness. The aspects of treatment effectiveness included the acquisition of speech and language skills (parents: mean 2.47, SD 0.92; 95% CI 2.27-2.67; students: mean 2.46, SD 0.93; 95% CI 2.17-2.76), communication with speech-language pathologists (parents: mean 2.52, SD 0.88; 95% CI 2.33-2.71; students: mean 2.32, SD 0.82; 95% CI 2.06-2.58), and treatment efficacy (parents: mean 2.33, SD 1.89; 95% CI 2.14-2.52; students: mean 2.37, SD 0.92; 95% CI 2.08-2.65). An independent 2-tailed sample *t* test revealed no significant differences in these aspects between parents and students (enjoyment: *P*=.41; acquisition of speech and language skills: *P*=.97; communication with speech-language pathologists: *P*=.22; treatment efficacy: *P*=.83). In addition, parents rated telepractice lower than on-site practice, in terms of the implementation of therapy practices at home via telepractice services or on-site services (mean 2.46; 95% CI 2.27-2.65).

Parents had a significant negative view of telepractice, with regard to whether they preferred telepractice over on-site practice (mean 2.24; 95% CI 2.04-2.43), whereas students had a neutral view (mean 3.07; 95% CI 2.74-3.41). An independent 2-tailed sample *t* test revealed a significant difference in preferences for telepractice and on-site practice between parents and students (t_124_=4.59; *P*<.001; *d*=0.87; 95% CI 0.48-1.26).

### Factors That Affected Preferences for Telepractice and On-site Practice

#### Grade

Our Spearman rank-order correlation analysis showed no significant correlations between student grade and participants’ preferences for the 2 service delivery modes (parents: ρ=0.07; *P=*.52; students: ρ=0.03; *P=*.85). The BF analysis showed strong evidence that supported the null hypothesis (ie, no correlation between grade and preferences for the mode of practice) in the parent group (BF_01_=10.89), whereas moderate evidence that supported the null hypothesis was found in the student group (BF_01_=8.17).

#### Treatment Efficacy

To examine the relationship between treatment efficacy and preferences for the 2 service delivery modes, we created a composite score based on the factor scores that were obtained from our exploratory factor analysis, by performing principal axis factoring extraction. As shown in Table 1, we obtained a factor score that accounted for 73% and 69% of the variance in the parent and student groups, respectively. All factor loadings were greater than .55.

**Table 1:** Principal axis factoring analysis of questions on telepractice efficacy. The pattern matrix for parents and students is shown.

Item	Parents^a^, factor loading value	Students^b^, factor loading value
Student enjoyment	.857	.552
Understanding of treatment goals	.798	.941
Meeting the needs of students	.926	.776
Enhancing speech and language abilities	.903	.819
Understanding treatment progress	.914	N/A^c^
Appropriate session frequency	.726	N/A
Appropriate session duration	.670	N/A

^a^The factor score for the parent group accounted for 73% of the variance in the items. Each item had an eigenvalue of 5.13.

^b^The factor score for the student group accounted for 69% of the variance in the items. Each item had an eigenvalue of 2.79.

^c^N/A: not applicable. These items only appeared in the parent questionnaire.

The Kendall rank correlation coefficient, τ, was computed based on the factor scores for telepractice efficacy and preferences for the mode of practice. No significant correlation was found in the parent group (τ=.07; *P=*.44); the BF for this correlation (BF_01_=8.53) moderately supported the null hypothesis (ie, there is no correlation between telepractice efficacy and preferences for the mode of practice). A significant correlation between telepractice efficacy and preferences for the mode of practice was found in the student group (τ=.43; *P*<.001).

## Discussion

### Principal Findings

Unlike previous telepractice studies, which have largely focused on clinicians’ attitudes, our study examined parents’ and students’ perceptions of telepractice efficacy and their attitudes toward telepractice during the COVID-19 pandemic. We found that students and parents were satisfied with the efficacy of treatments that were provided through telepractice services. Although students and parents had similar preferences for telepractice and on-site practice, parents preferred on-site practices. These findings are discussed in terms of telepractice efficacy and factors that affect engagement with telepractice services.

### Perceived Efficacy of Telepractice

One important finding of this study was that students and parents who engaged in telepractice services expressed satisfaction with these services, as evidenced by their ratings for telepractice services in school settings. These ratings show that telepractice services not only improved students’ speech and language abilities, but also increased students’ engagement with speech-language therapy and their motivations for learning. These results extend the findings of client satisfaction studies that focused on the evaluation of telepractice treatment programs [16,26,27]. These results also suggest that telepractice services help with retaining user satisfaction in real-life school service settings. Users’ satisfaction with telepractice is supported by compelling evidence concerning telepractice services for school-aged students with various disorders [7,28,29]. This evidence suggests that students with special education needs can benefit from treatments that are provided through telepractice services.

### Preference for Telepractice and On-site Practice

Despite students’ and parents’ satisfaction with telepractice efficacy, students did not prefer one mode of practice over the other, whereas parents preferred on-site practice over telepractice. However, there was no significant correlation between telepractice efficacy and parents’ preference for on-site practice (*P*=.44). This indicates that other concerns may have influenced parents’ preferences. Interestingly, compared to parents’ views of on-site practice, parents expressed a negative view of telepractice in terms of treatment effectiveness, the implementation of therapy practices at home, and communication with speech-language pathologists. This negative opinion can be explained by the lack of effective communication in telehealth. Due to the lack of personal interaction that occurs in telehealth services, extra communication and visual features for communication are needed to build a rapport between clinicians and parents [29]. For example, when discussing sensitive topics (eg, diagnosis, comorbidity, and prognosis) on web-based platforms, parents may feel a sense of depersonalization [29,30]. In addition, face-to-face communication has been indicated as a preferred mode of communication in various studies, as face-to-face communication allows for the better observation of visual cues, such as facial expressions and body language [31-33]. Another explanation for parents preferring on-site practices over telepractice is that parents need to provide extra effort and input in telepractice services. In telepractice sessions, parents need to solve technological problems and control students’ behaviors throughout the session. Therefore, parents must allocate more time and energy in telepractice sessions than they do in on-site sessions [33,34].

In this study, the students did not prefer one mode of practice over the other. This could be explained by their satisfaction with telepractice and the significant correlation between their perceptions of telepractice efficacy and their preferences for modes of practice (*P*<.001). Given that the students had fewer practical concerns than parents, and the fact that students acknowledged the effectiveness of both on-site practice and telepractice, they did not have a preference for the 2 service delivery modes.

Our findings also show that student grade was not significantly associated with telepractice efficacy (parents: *P*=.76; students: *P*=.65) or preferences for telepractice and on-site practice (parents: *P*=.52; students: *P*=.85). These results reflect the efficacy of telepractice and show that preferences did not differ considerably across different ages. This is consistent with other scientific studies, which have suggested that telepractice is suitable for school-aged students [7-9].

### Study Strengths

To our knowledge, this study is the first to investigate parents’ and students’ satisfaction with telepractice services for a school-aged population during the COVID-19 pandemic. Evaluating parents’ and students’ perceptions of the efficacy of telepractice is critical. This information not only helps speech-language therapists understand clients’ perceptions of telepractice, but also informs educational policy makers about the implementation and adoption of telepractice services beyond the pandemic period. Our study clearly demonstrates that users’ satisfaction with telepractice helps to promote evidence-based telepractice. Based on our analysis of both parents’ and students’ attitudes toward telepractice, we believe that both stakeholders acknowledged the efficacy of telepractice. This is a positive indicator for the future adoption of telepractice as another possible service delivery method, which is needed due to the potential psychosocial challenges of the COVID-19 pandemic. Such challenges include disrupted clinical routes, school closures, and reduced educational and medical support [35].

### Limitations and Future Research

This study focused on a limited sample size with a restricted age range (ie, Grades 1-7), even though school-based speech therapy services cover students in Grades 1-12. In addition, the small sample size restricted our investigation of the effect of comorbidity on telepractice efficacy, as communication and literacy characteristics can potentially affect telepractice efficacy.

Future research should consider investigating the effect of comorbidity on telepractice efficacy and satisfaction, by testing a larger sample that includes students of different ages and children with different types of special educational needs. For example, parental involvement is lower in the adolescent population than in the younger student population. Furthermore, in the adolescent population, treatment is focused on academic success. It is important to see whether the acceptance of telepractice services among adolescents differs from the acceptance among young, school-aged children. It should also be noted that our study focused on parents’ and students’ satisfaction with telepractice after a relatively short-term telepractice session. Future research should extend this study by investigating parents’ and students’ perceptions of telepractice efficacy and their attitudes toward telepractice after a long-term telepractice session. Our suggestions for future research may elucidate the long-term benefits and sustainability of telepractice, and provide guidance for telepractice strategy development. This information is needed to enhance the quality of digital medical approaches and psychological benefits for children and their families [36].

### Implications

The results of this study indicate that telepractice efficacy was well acknowledged by parents and students, and that students in Grades 1-7 had similar preferences for telepractice and on-site practice. The use of telepractice is supported not only by scientific evidence, but also by students’ and parents’ satisfaction. These results suggest that telepractice is a possible service delivery option for school-aged students.

The findings of our study are in line with those of existing literature, which suggests that telepractice is a suitable service delivery method [7-9]. Our study provided supporting evidence for schools and speech-language pathologists to adopt telepractice in real-life situations. In addition, our results suggest that speech-language pathologists and parents should be more proactive in telepractice services. Given that the parents had a negative view of treatment effectiveness and communication with speech-language pathologists during telepractice sessions, clinicians should consider engaging more effectively with both students and their parents. Speech-language pathologists can regularly update and inform parents and students about treatment effectiveness to increase their confidence during the transition to telepractice. In addition, clinicians should directly address parents’ concerns to build a therapeutic relationship [17]. The engagement and participation of parents is highly important in telepractice services. The importance of parent involvement is well noted in the literature [37,38], and the behavioral management of students during telepractice sessions relies on parents. Moreover, the role of the parent in telepractice services extends to providing technical support and troubleshooting [10]. Clinicians can pay attention to potential technical problems and provide relevant support to parents. If clinicians participate in and engage with telepractice services more often, it is expected that parents will have a better rapport with clinicians, which will facilitate the promotion and acceptance of telepractice [37].

### Conclusions

This study showed that both Hong Kong Chinese parents and students believed that telepractice was satisfactory and effective. Although students did not prefer one speech therapy delivery mode over the other, parents preferred on-site speech and language therapy. The perceived efficacy of telepractice was associated with students’ preferences for service delivery modes, but it was not associated with parents’ preferences. This could be explained by inadequate communication between clinicians and parents. Our findings suggest that it is necessary for speech-language pathologists to play a more proactive role by integrating telepractice into service delivery and explaining the efficacy of telepractice to parents and students.

## Appendix

Multimedia Appendix 1 Study questionnaire.

## Abbreviations

BF: Bayes factor

## References

[1] Camden C, Silva M (2021). Pediatric Teleheath: Opportunities Created by the COVID-19 and Suggestions to Sustain Its Use to Support Families of Children with Disabilities. Phys Occup Ther Pediatr.

[2] Wijesooriya NR, Mishra V, Brand PLP, Rubin BK (2020). COVID-19 and telehealth, education, and research adaptations. Paediatr Respir Rev.

[3] American Speech Language Hearing Association (2002). Survey report on telepractice use among audiologists and speech-language pathologists. American Speech Language Hearing Association.

[4] (2011). 2011 Membership Survey CCC-SLP Survey Summary Report: Number and Type of Responses. American Speech Language Hearing Association.

[5] Edwards M, Stredler-Brown A, Houston KT (2012). Expanding Use of Telepractice in Speech-Language Pathology and Audiology. Volta Rev.

[6] Mohan HS, Anjum A, Rao PKS (2017). A Survey of Telepractice in Speech-Language Pathology and Audiology in India. Int J Telerehabil.

[7] Boisvert M, Lang R, Andrianopoulos M, Boscardin ML (2010). Telepractice in the assessment and treatment of individuals with autism spectrum disorders: A systematic review. Dev Neurorehabil.

[8] Grogan-Johnson S, Gabel RM, Taylor J, Rowan LE, Alvares R, Schenker J (2011). A pilot exploration of speech sound disorder intervention delivered by telehealth to school-age children. Int J Telerehabil.

[9] Steele RD, Baird A, McCall D, Haynes L (2014). Combining Teletherapy and On-line Language Exercises in the Treatment of Chronic Aphasia: An Outcome Study. Int J Telerehabil.

[10] Telepractice. American Speech-Language-Hearing Association.

[11] Speech PA (2014). Position Statement Telepractice in speech pathology. Speech Pathology Asia.

[12] Fong R, Tsai CF, Yiu OY (2021). The Implementation of Telepractice in Speech Language Pathology in Hong Kong During the COVID-19 Pandemic. Telemed J E Health.

[13] Tohidast SA, Mansuri B, Bagheri R, Azimi H (2020). Determining pain in patients with voice disorders: a qualitative study. Logoped Phoniatr Vocol.

[14] Hines M, Lincoln M, Ramsden R, Martinovich J, Fairweather C (2015). Speech pathologists' perspectives on transitioning to telepractice: What factors promote acceptance?. J Telemed Telecare.

[15] Tucker JK (2012). Perspectives of speech-language pathologists on the use of telepractice in schools: quantitative survey results. Int J Telerehabil.

[16] Jessiman SM (2003). Speech and Language Services Using Telehealth Technology In Remote and Underserviced Areas. Canadian Journal of Speech-Language Pathology and Audiology.

[17] Fairweather GC, Lincoln MA, Ramsden R (2016). Speech-language pathology teletherapy in rural and remote educational settings: Decreasing service inequities. Int J Speech Lang Pathol.

[18] Bassi IB, Assunção AA, de Medeiros AM, de Menezes LN, Teixeira LC, Gama ACC (2011). Quality of life, self-perceived dysphonia, and diagnosed dysphonia through clinical tests in teachers. J Voice.

[19] Nelson LK, Masterson JJ (1999). Computer Technology: Creative Interfaces in Service Delivery. Top Lang Discord.

[20] Davis FD (1989). Perceived Usefulness, Perceived Ease of Use, and User Acceptance of Information Technology. MIS Quarterly.

[21] Moeller MP (2000). Early intervention and language development in children who are deaf and hard of hearing. Pediatrics.

[22] Basu S, Salisbury CL, Thorkildsen TA (2010). Measuring Collaborative Consultation Practices in Natural Environments. J Early Interv.

[23] Faul F, Erdfelder E, Lang AG, Buchner A (2007). G*Power 3: a flexible statistical power analysis program for the social, behavioral, and biomedical sciences. Behav Res Methods.

[24] de Winter JCF, Dodou DP, Wieringa PA (2009). Exploratory Factor Analysis With Small Sample Sizes. Multivariate Behav Res.

[25] Wetzels R, Wagenmakers EJ (2012). A default Bayesian hypothesis test for correlations and partial correlations. Psychon Bull Rev.

[26] Grogan-Johnson S, Alvares R, Rowan L, Creaghead N (2010). A pilot study comparing the effectiveness of speech language therapy provided by telemedicine with conventional on-site therapy. J Telemed Telecare.

[27] McGill M, Noureal N, Siegel J (2019). Telepractice Treatment of Stuttering: A Systematic Review. Telemed J E Health.

[28] Wales D, Skinner L, Hayman M (2017). The Efficacy of Telehealth-Delivered Speech and Language Intervention for Primary School-Age Children: A Systematic Review. Int J Telerehabil.

[29] Steindal SA, Nes AAG, Godskesen TE, Dihle A, Lind S, Winger A, Klarare A (2020). Patients' Experiences of Telehealth in Palliative Home Care: Scoping Review. J Med Internet Res.

[30] Weaver MS, Neumann ML, Navaneethan H, Robinson JE, Hinds PS (2020). Human Touch via Touchscreen: Rural Nurses' Experiential Perspectives on Telehealth Use in Pediatric Hospice Care. J Pain Symptom Manage.

[31] Marziali E, Damianakis T, Donahue P (2008). Internet-Based Clinical Services: Virtual Support Groups for Family Caregivers. J Technol Hum Serv.

[32] White M, Dorman SM (2001). Receiving social support online: implications for health education. Health Educ Res.

[33] Yoo J, Yoon M, Lee CK, Hong GH, Choi SJ (2020). An Exploratory Survey of Priorities in Establishing Telepractice System for SLPs and Caregivers in Korea. Commun Disord Q.

[34] Grillo EU (2017). Results of a Survey Offering Clinical Insights into Speech-Language Pathology Telepractice Methods. Int J Telerehabil.

[35] Badawy SM, Radovic A (2020). Digital Approaches to Remote Pediatric Health Care Delivery During the COVID-19 Pandemic: Existing Evidence and a Call for Further Research. JMIR Pediatr Parent.

[36] Serlachius A, Badawy SM, Thabrew H (2020). Psychosocial Challenges and Opportunities for Youth With Chronic Health Conditions During the COVID-19 Pandemic. JMIR Pediatr Parent.

[37] Bowen C, Cupples L (2004). The role of families in optimizing phonological therapy outcomes. Child Lang Teach Ther.

[38] Tambyraja SR, Schmitt MB, Farquharson K, Justice LM (2017). Home literacy environment profiles of children with language impairment: associations with caregiver- and child-specific factors. Int J Lang Commun Disord.

