# Supplementary material for: Parents’ and Students’ Perceptions of Telepractice Services for Speech-Language Therapy During the COVID-19 Pandemic: Survey Study
Source: JMIR Pediatr Parent. 2021 Jan 28;4(1):e25675. doi: 10.2196/25675 (PMC7850632; doi:10.2196/25675)
Supplement: Multimedia Appendix 1 [file pediatrics_v4i1e25675_app1.docx]

## Multimedia Appendix 1.

### Study Questionnaire.

#### Section A. Telepractice Implementation.

1. How many telepractice session(s) did your child (you) receive during the pandemic?

__ 1 to 5 session(s)

__ 6 to 10 sessions

__ more than 10 sessions

1. Please estimate the frequency of the telepractice your child (you) received.

__ weekly

__ biweekly

__ monthly

__ less than monthly

#### Section B. Telepractice Efficacy.

Please rate the statements below, regarding telepractice, on a scale of 1 to 5. (1 = Strongly disagree; 2 = Disagree; 3 = Neutral; 4 = Agree; 5 = Strongly agree)

1. __ My child (I) enjoyed telepractice.
2. __ I understood the telepractice training aims.
3. __ The training aims met the needs of my child (me).
4. __ Telepractice was effective in enhancing the speech and language abilities of my child (me).
5. *__ I understood the treatment progress during telepractice.
6. *__ The telepractice treatment frequency was appropriate.
7. *__ The telepractice treatment duration was appropriate.

#### Section C. Comparison of Telepractice and Onsite Practice.

Please rate the statements below, on a scale of 1 to 5 (1 = Strongly disagree; 2 = Disagree; 3 = Neutral; 4 = Agree; 5 = Strongly agree).

1. __ My child (I) like telepractice more than onsite practice.
2. __ I learn language skills better in telepractice than in onsite practice.
3. *__ I learn how to carry out home practice better in telepractice than in onsite practice.
4. __ I have better communication with the speech-language pathologist in telepractice than in onsite practice.
5. __ The treatment efficacy is higher in telepractice than in onsite practice.
6. __ I prefer telepractice to onsite practice.

#### Section D. Demographics. (To be filled in by caregivers in both versions).

1. Grade __. Please state the grade that your child is in.

__ Please state the gender of your child (M/F).

1. Please indicate the special education needs your child.

__ Attention deficit/hyperactive disorder

__ Autism spectrum disorder

__ Hearing impairment

__ Intellectual disability

__ Mental illness

__ Physical disability

__ Specific learning disorder (including dyslexia)

__ Visual impairment

1. Please indicate your average monthly family income in the first half of 2020.

__ < HKD10,000

__ HKD10,001 – 20,000

__ HKD10,001 – 30,000

__ HKD10,001 – 40,000

__ HKD10,001 – 50,000

__ HKD10,001 – 60,000

__ HKD10,001 – 70,000

__ HKD10,001 – 80,000

__ HKD10,001 – 90,000

__ HKD10,001 – 100,000

__ > HKD100,001

Note. The pronouns in brackets () are used in the students’ version. The questions marked with an asterisk (*) are only in the caregivers’ version.
